# Circadian Disruption and Breast Cancer Risk: Evidence from a Case-Control Study in China

**DOI:** 10.3390/cancers15020419

**Published:** 2023-01-08

**Authors:** Song Song, Lin Lei, Rui Zhang, Han Liu, Jia Du, Ni Li, Wanqing Chen, Ji Peng, Jiansong Ren

**Affiliations:** 1 Office of Cancer Screening, National Cancer Center/National Clinical Research Center for Cancer/Cancer Hospital, Chinese Academy of Medical Sciences and Peking Union Medical College, Beijing 100021, China; 2 Department of Cancer Control and Prevention, Shenzhen Center for Chronic Disease Control, Shenzhen 518000, China; 3 Office of Cancer Prevention and Control, Chongqing University Cancer Hospital, Chongqing 400030, China; 4 Key Laboratory of Big Data Analysis and Implement, Chinese Academy of Medical Sciences, Beijing 100021, China

**Keywords:** breast cancer, circadian disruption, night shift work, light exposure at night, sleep duration, circadian gene

## Abstract

**Simple Summary:**

Rare studies had been conducted in the Chinese population regarding the impact of circadian disruptors on breast cancer risk. This study found that a short duration of sleep was significantly associated with breast cancer. Besides, rs2292912 in *CRY2*, rs2253820 in *PER1*, rs2289591 in *PER1* and rs3027188 in *PER1* were positively associated with the risk of breast cancer.

**Abstract:**

Studies had suggested an association between circadian disruptors (including night shift work, domestic light exposure at night, sleep duration, and circadian gene polymorphism) and breast cancer, while rare studies had been conducted in the Chinese population. This study was a case-control study conducted to explore the impact of circadian disruptors on the risk of breast cancer in China. Four hundred and sixty-four cases and 464 controls, admitted from the Department of Breast Surgery, Cancer Hospital, Chinese Academy of Medical Sciences, were included in this study. Adjusting age, BMI group, smoking, alcohol consumption, menopausal status, family history of breast cancer, duration of breastfeeding, age at menarche, number of pregnancies, age at first full-term pregnancy, use of estrogen and use of oral contraceptive, multivariate logistic regression analysis showed that the risk of breast cancer was higher in short sleep duration group (OR = 4.86, 95%CI: 1.73–17.33). Meanwhile, rs2292912 in *CRY2*, rs2253820 in *PER1*, rs2289591 in *PER1* and rs3027188 in *PER1* were positively associated with the risk of breast cancer. This study supported that the short duration of sleep and four SNPs in crucial circadian genes played a role in the development of breast cancer.

## 1. Introduction

GLOBOCAN 2020 [1] estimated that about 2.26 million new breast cancer cases and 685 thousand breast cancer deaths occurred in 2020 globally. The National Cancer Center of China [2] estimated that 306 thousand new breast cancer cases and 71.7 thousand breast cancer deaths occurred in 2016 in China. Breast cancer had become one of the major public health problems in China. Currently known risk factors (e.g., aging, family history, reproductive factors, estrogen, lifestyle, etc.) can only explain part of the breast cancer cases (23.20–55.95%), and there are still unknown risk factors accounting for the occurrence of breast cancer [3,4,5].

More and more evidence showed that there was an association between circadian rhythm disruption and cancer risk [6,7,8]. Circadian disruption may adversely affect many physiologic functions (e.g., respiration, metabolism and immune system) and biological activities (e.g., hormonal secretions, apoptosis and DNA repair) [7,9,10]. In 2019, the International Agency for Research on Cancer (IARC) confirmed that night shift work (work during the regular sleeping hours of the general population) was a probable carcinogen to humans (Group 2A) based on limited epidemiological research and extensive animal studies, and circadian disruption is the most important effect [11]. There are some studies investigating the association between night shift work and breast cancer, but the results are inconsistent. A recent meta-analysis of 57 observational studies failed to show an association between night-shift work and breast cancer [12]. The systematic review by Manouchehri found that the relative risk (RR) of breast cancer was 1.13 (95%CI: 1.03–1.24) in the short-term night-shift workers (<10 years), while no positive statistical association was found in the long-term night-shift workers (≥10 years) [13].

Besides, only light exposure at night, caused by night shift work, domestic light at night (e.g., outdoor light and indoor light while sleeping), and so on, can affect circadian rhythm [14,15]. Since 1960, several animal experiments [16,17,18] had shown that melatonin concentration in rats showed a distinct circadian rhythm, with significantly higher concentrations at night than daytime. Light exposure at night, even for a minute, would result in a dramatic decrease in melatonin concentration in rats [19]. In 1980, Lewy et al. [20] first reported that melatonin level in human was related to light intensity. Melatonin is mainly produced by the pineal gland, and its secretion has a circadian rhythm with more secretion at night, but light exposure at night may inhibit melatonin secretion and then promote the secretion of female hormones, which consequently increases the risk of breast cancer [21]. While limited studies are currently focused on occupational populations, and fewer studies have explored the association between domestic light at night and breast cancer. In addition to the above, findings have shown that there may be an association between sleep duration and breast cancer, while some other studies did not [22,23,24,25].

Circadian rhythms are the result of complex physiological processes in which the external and internal environments interact, and these physiological processes are regulated by a set of circadian genes [26]. In particular, core circadian genes play an important role in tumor-related biological mechanisms such as cell cycle regulation, DNA repair, and apoptosis [27]. Polymorphisms in circadian genes have been found to be significantly associated with the risk of breast cancer [28,29].

Several studies had suggested an association between circadian disruptors (including night shift work, domestic light exposure at night, sleep duration, and circadian gene polymorphism) and breast cancer risk [14,25,30,31]. However, to date, studies had rarely been reported to explore the impact of circadian disruptors on the risk of breast cancer in the Chinese population. Therefore, this study was conducted to further clarify the association between circadian disruption and breast cancer risk.

## 2. Materials and Methods

### 2.1. Study Population

Using an unmatched case-control study design, participants were all obtained from the Department of Breast Surgery, Cancer Hospital, Chinese Academy of Medical Sciences between July 2011 and December 2012. Participants were eligible if: (I) female; (II) consented to participate in this study and completed the questionnaire survey. Patients of previously diagnosed malignant tumor, non-primary breast cancer, and recurrent breast cancer were excluded from this study. In the end, 464 newly diagnosed breast cancer patients were collected as the case group. The control group was 464 histologically-confirmed benign breast disease patients collected from the same department during the same period.

### 2.2. Data Collection

Before the survey, we got informed consent from each participant. All participants were then interviewed face-to-face with well-trained investigators using a structured questionnaire. This questionnaire covered demographic characteristics (e.g., age, height, weight, and education level), personal behavior (e.g., cigarette smoking and alcohol drinking), history of disease, family history of breast cancer, use of estrogenic drugs, night work, shift work, domestic light exposure at night (e.g., outdoor light and indoor light while sleeping) and sleep duration. 

Based on the guidelines for the prevention and control of overweight and obesity in adults in China [32], body mass index (BMI) was categorized into three groups: low and normal weight (<24 kg/m^2^), overweight (24.0 to 27.9 kg/m^2^) and obesity (≥28.0 kg/m^2^). Cigarette smoking was defined as cumulative smoking greater than 100 cigarettes. Alcohol consumption refers to drinking alcohol at least once a week for more than six months. Menopause refers to the absence of menstruation for six consecutive months or more. Family history of breast cancer refers to having more than one first-degree relative (parents, brothers, sisters, and children) who was diagnosed with breast cancer. Night work was defined as ever having worked between midnight and 5 a.m. for more than one year, and shift work was defined as ever having worked at night, or on night shifts, or on call at night for more than one year.

### 2.3. Selection of SNPs

According to National Center for Biotechnology Information (NCBI) gene and dbSNP database (https://www.ncbi.nlm.nih.gov/, (accessed on 1 January 2016)) and published articles [8,29,30,33,34,35], we considered the functional SNPs in circadian genes as well as the association between night work, shift work, circadian genes, and breast cancer risk. In the end, 27 SNPs of 7 crucial circadian genes (*CLOCK*, *CRY1*, *CRY2*, *NPAS2*, *PER1*, *PER2,* and *TIMELESS*) were selected for genotyping. The basic information of 27 candidate SNPs is shown in Table S1.

### 2.4. Samples Collection and Laboratory Test

The blood samples (5 mL) were collected from all participants and stored at −80 °C. Using blood samples acquired, DNA was extracted using the Genomic DNA Extraction Kit developed by Berry Genomics (Beijing, China) and stored at −20 °C. Cut the neighboring sequence of candidate SNPS, and primers were then designed using MassARRAY Assay Design software Version 3.1 (Sequenom, San Diego, CA, USA). Then the circadian gene polymorphisms were genotyped by the Sequenom MassARRAY system (Sequenom, San Diego, CA, USA) through amplification polymerase chain reaction, shrimp alkaline phosphatase reaction, and primer extension reaction. 

### 2.5. Statistical Analysis

The characteristics of participants were described as percentages and compared with the χ^2^ test for categorical variables, and the *t*-test for continuous variables. Association between circadian disruptors and breast cancer were assessed by non-conditional logistic regression with odds ratios (OR) and their 95% confidence intervals (CIs). Using codominant, dominant, and recessive genetic models, 24 of 27 SNPs with a minor allele frequency (MAF) > 0.01, and a Hardy–Weinberg equilibrium (HWE) *p*-value > 0.001 were included in logistic regression to measure the association between circadian gene polymorphism and breast cancer risk. EpiData 3.1 was used for questionnaire entry and logical verification. All the statistical analysis was performed with R 4.2.1. All tests were two-sided and the statistical significance level was set at *p* < 0.05.

## 3. Results

### 3.1. Basic Characteristics of Study Population

The basic characteristics of the study population were detailed in Table 1. Among 464 cases and 464 controls, the mean age was 44.64 (±11.35) and 45.13 (±10.53) years old respectively. The majority of participants were nonsmokers (96.5%) and nondrinkers (90.0%). Overall, 31.2% of participants reported menopause and 6.9% had a family history of breast cancer. The distribution of use of oral contraceptives was significantly different between the two groups. 

### 3.2. Night Shift Work, Domestic Light at Night, Sleep Duration, and Breast Cancer Risk

Based on previous studies, age, BMI group, smoking, alcohol consumption, menopausal status, family history of breast cancer, duration of breastfeeding, age at menarche, number of pregnancies, age at first full-term pregnancy, use of estrogen and use of oral contraceptive were included in the multivariate logistic regression analysis. It showed that night work and shift work had no association with breast cancer (Table 2). Women not pulling curtains during sleep may have a higher risk of breast cancer (OR = 1.15, 95%CI: 0.78–1.71). The adjusted OR of women who sleep with the lights on was 1.26 (95%CI: 0.67–2.41). While there was no statistically significant association between domestic light at night and breast cancer. The short duration of sleep (OR = 4.86, 95%CI: 1.73–17.33) was a significant risk factor for breast cancer, and long sleep duration (OR = 1.16, 95%CI: 0.88–1.53) may be associated with breast cancer.

### 3.3. SNPs and Breast Cancer Risk

Univariate analyses of three genetic models (codominant, dominant, and recessive) were performed for each SNP (Table S2). SNPs with statistically significant results were included in subsequent analyses (Table 3). Compared to the GG genotype of *CRY2* rs2292912, the CC genotype was significantly more common in the cancer cases (OR = 1.71, 95%CI: 1.07–2.75). A significant association was shown between the GG genotype of rs2253820 in *PER1* and increased risk of breast cancer (OR = 1.61, 95%CI: 1.00–2.61). The OR of the *PER1* (rs2289591) GT genotype was 1.81 (95%CI: 1.05–3.19) compared with GG, and the OR of *PER1* (rs3027188) GG genotype was 1.67 (95%CI: 1.04–2.71) compared with CC.

## 4. Discussion

The current case-control study, involving 464 cases and 464 controls in China, found that lower sleep duration was significantly associated with higher breast cancer. Meanwhile, rs2292912 in *CRY2*, rs2253820 in *PER1*, rs2289591 in *PER1* and rs3027188 in *PER1* could significantly increase the risk of breast cancer. 

The present study showed that there was no statistically significant association between night work, shift work, and breast cancer. A meta-analysis, with 33 observational studies composed of 4,331,782 participants published before June 2021, found that night shift work showed a significant association with breast cancer risk [hazard ratio (HR) = 1.20, 95% Cl: 1.10–1.31] [31]. Using the cohorts of the Nurses’ Health Study and Nurses’ Health Study II, the study revealed that women would have a higher risk of breast cancer (HR = 1.40, 95%CI: 1.00–1.97) only if they had experienced shift work (work at least three nights/month) for 20 years or longer [36]. A case-control study of the Chinese population observed that night shift work (working at least once per week for at least 6 months between midnight and 6 a.m.) was associated with an increased risk of breast cancer (OR = 1.34, 95%CI: 1.05–1.72) [37]. While another nested case-control study, conducted in Shanghai, China, found no associations with either duration or frequency of night-shift work (working between 12 p.m. and 5 a.m.), which is consistent with our study [38]. This discrepancy may be the result of inconsistent definitions of night shift work across studies and the presence of recall bias or confounding factors.

We found that domestic light exposure at night may be a potential risk factor for breast cancer, while none of the results were statistically significant. In this study, women not pulling curtains (OR = 1.15, 95%CI: 0.78–1.71) and light on (OR = 1.26, 95%CI: 0.67–2.41) during sleep may have a higher risk of breast cancer. A case-control study in Connecticut conducted by Li et al. [39] found that turning on the light (OR = 1.4, 95%CI: 0.7–2.7) or not drawing the curtains/window shades (OR = 1.2, 95%CI: 0.8–1.9) during nighttime sleep may increase breast cancer risk, which is consistent with the results of our study. A case-control study by Kloog et al. [40] revealed that women who slept in a bright environment had a higher risk for breast cancer than those who slept in a dark environment (OR = 1.22, 95%CI: 1.12–1.31). A prospective cohort study including 10,500 UK women, after 6.1 years of follow-up, found that the HR of breast cancer was 1.01 (95%CI: 0.88–1.15) among those with a bright living environment at baseline compared to those with a dark living environment at sleep [41]. Our study found no relationship between breast cancer and bedroom brightness while sleeping, which may be caused by inconsistent classification compared with other studies, limited sample size, recall bias, or the existence of confounding factors.

We observed that short duration of sleep (OR = 4.86, 95%CI: 1.73–17.33) increased the risk of breast cancer, and long sleep duration (OR = 1.16, 95%CI: 0.88–1.53) may be a risk factor for breast cancer. The most recent meta-analyses published in 2021, including 15 prospective studies and 65,410 breast cancer cases, found that there was no association between short sleep duration (RR = 0.99, 95%CI: 0.98–1.01), long sleep duration (RR = 1.01, 95%CI: 0.98–1.04) and breast cancer [25]. A prospective cohort study including 23,995 Japanese women revealed that women who slept ≤ 6 h (HR = 1.62, 95%CI: 1.05–2.50) were at an increased risk of breast cancer than women whose duration of sleep was 7 h [42]. A prospective study of the Sister Study cohort (N = 50,884) found no association between sleep duration and breast cancer, while women having difficulty sleeping ≥ 4 nights a week had a higher risk for overall (HR = 1.32, 95% CI: 1.09–1.61) and postmenopausal breast cancer (HR = 1.51, 95% CI: 1.24–1.85) compared to those with no sleeping difficulty [43].

Our analysis showed that four SNPs, including rs2292912 in *CRY2*, rs2253820 in *PER1*, rs2289591 in *PER1* and rs3027188 in *PER1*, were significantly associated with breast cancer risk. A case-control study from Korea, with 941 cases and 959 controls, found that night-shift work could increase breast cancer risk in women carrying the CG genotype of rs2292912 in *CRY2* (OR = 2.03, 95%CI: 1.15–3.58) [29]. Rs2253820 in *PER1* (OR = 1.31, 95%CI: 1.17–1.48) was significantly associated with Parkinson’s disease risk in Han-nationality Chinese [44]. In a case-control study conducted in a Caucasian population, rs2289591 in *PER1* (OR = 1.25; 95%CI: 1.00–1.57) was associated with more aggressive prostate cancer risk under a dominant genetic model [45]. A case-control study from Canada, with 1054 cases and 1016 controls, found that there were no associations between rs3027188 in *PER1* and breast cancer risk [46].

The current study had several advantages. First of all, our study collected detailed information about domestic light exposure at night, such as bedroom brightness and outdoor light while sleeping. Second, we explored the association between light exposure at night in occupational settings and domestic settings, which was comprehensive. Meanwhile, several limitations of this study need to be acknowledged. First, the sample size of this study was limited. Furthermore, it was difficult for people to accurately report their light exposure at night because of recall bias, resulting in unavoidable misclassification of circadian disruption. At last, due to the lack of relevant data, we were unable to perform a prognostic analysis on the overall survival of patients.

## 5. Conclusions

In summary, the present study conducted on Chinese women suggested that a short duration of sleep and four SNPs in crucial circadian genes played a role in the development of breast cancer. More epidemiological studies need to be implemented in a larger population to further explore the relationship between circadian disruption and breast cancer.

## Figures and Tables

**Table 1:** Basic characteristics of study population.

Characteristics	Overall (N = 928)	Control (N = 464)	Case (N = 464)	P ^1^
Age (years)				0.496
Mean age	44.89 ± 10.95	44.64 ± 11.35	45.13 ±10.53	
Age group (years)				0.999
<40	278 (30.0)	139 (30.0)	139 (30.0)	
40~49	392 (42.2)	195 (42.0)	197 (42.5)	
50~59	167 (18.0)	84 (18.1)	83 (17.9)	
≥60	91 (9.8)	46 (9.9)	45 (9.7)	
BMI group (kg/m^2^)				0.195
≤23.9	518 (56.1)	259 (56.4)	259 (55.8)	
24.0–27.9	285 (30.9)	149 (32.5)	136 (29.3)	
≥28.0	120 (13.0)	51 (11.1)	69 (14.9)	
Smoking				0.450
Never	899 (96.9)	452 (97.4)	447 (96.3)	
Ever	29 (3.1)	12 (2.6)	17 (3.7)	
Alcohol consumption				0.058
Never	837 (90.3)	428 (92.2)	409 (88.3)	
Ever	90 (9.7)	36 (7.8)	54 (11.7)	
Menopausal status				0.957
Pre-menopause	673 (72.6)	337 (72.8)	336 (72.4)	
Post-menopause	254 (27.4)	126 (27.2)	128 (27.6)	
Family history of breast cancer				0.330
No	873 (94.2)	440 (95.0)	433 (93.3)	
Yes	54 (5.8)	23 (5.0)	31 (6.7)	
Duration of breastfeeding (months)				0.303
Never	230 (25.5)	120 (27.1)	110 (24.0)	
0~6	118 (13.1)	62 (14.0)	56 (12.2)	
>6	553 (61.4)	260 (58.8)	293 (63.8)	
Age at menarche (years)				0.901
≤14	518 (55.9)	257 (55.6)	261 (56.2)	
>14	408 (44.1)	205 (44.4)	203 (43.8)	
Number of pregnancies				0.139
Nullipara	75 (8.1)	45 (9.8)	30 (6.5)	
1~2	438 (47.5)	219 (47.7)	219 (47.2)	
≥3	410 (44.4)	195 (42.5)	215 (46.3)	
Age at first full-term pregnancy (years)				0.261
Nullipara	119 (12.8)	67 (14.4)	52 (11.2)	
<28	584 (62.9)	282 (60.8)	302 (65.1)	
≥28	225 (24.2)	115 (24.8)	110 (23.7)	
Use of estrogen				0.246
Never	817 (90.5)	397 (89.2)	420 (91.7)	
Ever	86 (9.5)	48 (10.8)	38 (8.3)	
Use of oral contraceptive				0.047
Never	741 (81.9)	354 (79.2)	387 (84.5)	
Ever	164 (18.1)	93 (20.8)	71 (15.5)	

^1^*p*-value of the chi-square test for categorical variables and *t*-test for continuous variables. Abbreviation: SD: standard difference; BMI: body mass index.

**Table 2:** Association between night shift work, domestic light at night, sleep duration, and breast cancer risk in univariate and multivariate logistic regression analysis.

Circadian Disruptor	Level	Overall (N = 928)	Control (N = 464)	Case (N = 464)	OR (95%CI)	Adjusted OR ^1^ (95%CI)
Night work experience	Never	734 (79.1)	360 (77.6)	374 (80.6)	Reference	Reference
	Ever	194 (20.9)	104 (22.4)	90 (19.4)	0.83 (0.61–1.14)	0.84 (0.60–1.17)
Duration of night work	Never	734 (79.4)	360 (77.9)	374 (80.8)	Reference	Reference
(years)	<10	107 (11.6)	59 (12.8)	48 (10.4)	0.78 (0.52–1.18)	0.75 (0.49–1.14)
	≥10	84 (9.1)	43 (9.3)	41 (8.9)	0.92 (0.58–1.44)	0.96 (0.60–1.56)
Frequency of night work	Never	734 (79.5)	360 (78.1)	374 (81.0)	Reference	Reference
(times/month)	<10	115 (12.5)	63 (13.7)	52 (11.3)	0.79 (0.53–1.18)	0.81 (0.54–1.22)
	≥10	74 (8.0)	38 (8.2)	36 (7.8)	0.91 (0.56–1.47)	0.90 (0.55–1.48)
Shift work experience	Never	700 (77.3)	337 (75.2)	363 (79.4)	Reference	Reference
	Ever	205 (22.7)	111 (24.8)	94 (20.6)	0.79 (0.57–1.07)	0.81 (0.58–1.13)
Duration of shift work	Never	700 (77.7)	337 (75.6)	363 (79.8)	Reference	Reference
(years)	<10	108 (12.0)	61 (13.7)	47 (10.3)	0.72 (0.47–1.07)	0.72 (0.47–1.09)
	≥10	93 (10.3)	48 (10.8)	45 (9.9)	0.87 (0.56–1.34)	0.94 (0.59–1.50)
Frequency of shift work	Never	700 (78.0)	337 (76.1)	363 (79.8)	Reference	Reference
(times/month)	<10	74 (8.2)	43 (9.7)	31 (6.8)	0.67 (0.41–1.08)	0.69 (0.41–1.13)
	≥10	124 (13.8)	63 (14.2)	61 (13.4)	0.90 (0.61–1.32)	0.91 (0.61–1.35)
Curtain pulling while	Yes	794 (86.0)	400 (87.0)	394 (85.1)	Reference	Reference
sleeping	No	129 (14.0)	60 (13.0)	69 (14.9)	1.17 (0.80–1.70)	1.15 (0.78–1.71)
Light on while	No	876 (94.9)	440 (95.7)	436 (94.2)	Reference	Reference
sleeping	Yes	47 (5.1)	20 (4.3)	27 (5.8)	1.36 (0.76–2.49)	1.26 (0.67–2.41)
Bedroom brightness	None	450 (49.1)	216 (47.6)	234 (50.5)	Reference	Reference
while sleeping	Low	391 (42.6)	198 (43.6)	193 (41.7)	0.90 (0.69–1.18)	0.85 (0.64–1.13)
	Average	62 (6.8)	31 (6.8)	31 (6.7)	0.92 (0.54–1.57)	1.00 (0.57–1.74)
	High	14 (1.5)	9 (2.0)	5 (1.1)	0.51 (0.16–1.51)	0.47 (0.13–1.47)
Outdoor light	No	664 (72.7)	329 (72.8)	335 (72.7)	Reference	Reference
while sleeping	Yes	249 (27.3)	123 (27.2)	126 (27.3)	1.01 (0.75–1.35)	1.01 (0.74–1.36)
Duration of sleep	6–8 h	483 (52.9)	252 (55.5)	231 (50.3)	Reference	Reference
	<6 h	24 (2.6)	5 (1.1)	19 (4.1)	4.15 (1.64–12.66)	4.86 (1.73–17.33)
	>8 h	406 (44.5)	197 (43.4)	209 (45.5)	1.16 (0.89–1.51)	1.16 (0.88–1.53)

^1^ OR adjusted by age, BMI group, smoking, Alcohol consumption, menopausal status, family history of breast cancer, duration of breastfeeding, age at menarche, number of pregnancies, age at first full-term pregnancy, use of estrogen, use of oral contraceptive.

**Table 3:** Association between SNPs of circadian genes with breast cancer risk in univariate and multivariate logistic regression analysis.

SNP	Genotype	Total (N = 928)	Control (N = 464)	Case (N = 464)	OR (95%CI)	Adjusted OR ^1^ (95%CI)
*CRY1* rs10778527	TT	463 (54.2)	210 (51.3)	253 (56.9)	Reference	Reference
	TC	342 (40.0)	180 (44.0)	162 (36.4)	0.75 (0.56–0.99)	0.75 (0.56–1.01)
	CC	49 (5.7)	19 (4.6)	30 (6.7)	1.31 (0.72–2.43)	1.17 (0.64–2.21)
	TC + CC ^2^	391 (45.8)	199 (48.7)	192 (43.1)	0.80 (0.61–1.05)	0.80 (0.60–1.05)
	CC ^3^	49 (5.7)	19 (4.6)	30 (6.7)	1.48 (0.83–2.72)	1.33 (0.73–2.47)
*CRY2* rs2292912	GG	351 (39.3)	175 (39.7)	176 (39.0)	Reference	Reference
	GC	432 (48.4)	224 (50.8)	208 (46.1)	0.92 (0.70–1.22)	0.88 (0.66–1.18)
	CC	109 (12.2)	42 (9.5)	67 (14.9)	1.59 (1.03–2.47)	1.71 (1.07–2.75)
	GC + CC ^2^	541 (60.7)	266 (60.3)	275 (61.0)	1.03 (0.79–1.34)	1.00 (0.76–1.33)
	CC ^3^	109 (12.2)	42 (9.5)	67 (14.9)	1.66 (1.10–2.51)	1.83 (1.18–2.86)
*PER1* rs2253820	AA	439 (49.6)	229 (52.3)	210 (47.0)	Reference	Reference
	AG	355 (40.1)	174 (39.7)	181 (40.5)	1.13 (0.86–1.5)	1.09 (0.82–1.47)
	GG	91 (10.3)	35 (8.0)	56 (12.5)	1.74 (1.10–2.79)	1.61 (1.00–2.61)
	AG + GG ^2^	446 (50.4)	209 (47.7)	237 (53.0)	1.24 (0.95–1.61)	1.18 (0.90–1.56)
	GG ^3^	91 (10.3)	35 (8.0)	56 (12.5)	1.65 (1.06–2.59)	1.55 (0.98–2.46)
*PER1* rs2289591	GG	806 (92.4)	406 (94.4)	400 (90.5)	Reference	Reference
	GT	63 (7.2)	22 (5.1)	41 (9.3)	1.89 (1.12–3.28)	1.81 (1.05–3.19)
	TT	3 (0.3)	2 (0.5)	1 (0.2)	0.51 (0.02–5.32)	0.55 (0.02–6.00)
	GT + TT ^2^	66 (7.6)	24 (5.6)	42 (9.5)	1.78 (1.06–3.03)	1.71 (1.01–2.96)
	TT ^3^	3 (0.3)	2 (0.5)	1 (0.2)	0.49 (0.02–5.08)	0.52 (0.02–5.67)
*PER1* rs3027188	CC	448 (50.3)	232 (53.0)	216 (47.8)	Reference	Reference
	CG	352 (39.6)	172 (39.3)	180 (39.8)	1.12 (0.85–1.49)	1.09 (0.81–1.46)
	GG	90 (10.1)	34 (7.8)	56 (12.4)	1.77 (1.12–2.84)	1.67 (1.04–2.71)
	CG + GG ^2^	442 (49.7)	206 (47.0)	236 (52.2)	1.23 (0.95–1.60)	1.19 (0.90–1.57)
	GG ^3^	90 (10.1)	34 (7.8)	56 (12.4)	1.68 (1.08–2.65)	1.60 (1.01–2.56)
*PER2* rs2304674	TT	413 (48.2)	188 (45.1)	225 (51.1)	Reference	Reference
	TC	385 (44.9)	194 (46.5)	191 (43.4)	0.82 (0.62–1.09)	0.79 (0.59–1.05)
	CC	59 (6.9)	35 (8.4)	24 (5.5)	0.57 (0.33–0.99)	0.60 (0.34–1.07)
	TC + CC ^2^	444 (51.8)	229 (54.9)	215 (48.9)	0.78 (0.60–1.03)	0.76 (0.57–1.01)
	CC ^3^	59 (6.9)	35 (8.4)	24 (5.5)	0.63 (0.36–1.07)	0.68 (0.38–1.17)

^1^ OR adjusted by age, BMI group, smoking, Alcohol consumption, menopausal status, family history of breast cancer, duration of breastfeeding, age at menarche, number of pregnancies, age at first full-term pregnancy, use of estrogen, use of oral contraceptive. ^2^ A dominant genetic model. ^3^ A recessive genetic model.

## Data Availability

Data analysis and management are only permitted within Cancer Hospital, Chinese Academy of Medical Sciences in China. Further information is available from the corresponding author on reasonable request.

## Supplementary Materials

The following supporting information can be downloaded at: https://www.mdpi.com/article/10.3390/cancers15020419/s1, Table S1: Basic information of selected circadian genes’ 27 candidate SNPs; Table S2: Univariate logistic regression to explore the relationship between SNPs and breast cancer.
